# Pulmonary Rehabilitation Outcomes of Post-Acute COVID-19 Patients during Different Waves of the Pandemic

**DOI:** 10.3390/ijerph20105907

**Published:** 2023-05-22

**Authors:** Marc Spielmanns, Corina E. Schaer, Anna-Maria Pekacka-Egli, Sabine Spielmanns, Olberk Ibish, Guzel Gafina, Antonela Stiube, Matthias Hermann

**Affiliations:** 1 Pulmonary Medicine, Zuercher RehaZentren, Klinik Wald, 8636 Wald, Switzerland; corina.schaer@zhreha.ch (C.E.S.);; 2 Department for Pulmonary Medicine, Faculty of Health, University Witten Herdecke, 58455 Witten, Germany; 3 Department of Cardiology, University Heart Center, University Hospital Zurich, 8006 Zurich, Switzerland

**Keywords:** COVID-19 waves, 6-MWT, FIM, CIRS, pandemic, pulmonary rehabilitation

## Abstract

(1) Background: Between the beginning of the coronavirus pandemic and summer 2022, we distinguished four pandemic waves, with different characteristics of the affected patients. This study investigated the impact of patient characteristics on the outcome of inpatient pulmonary rehabilitation (PR). (2) Methods: Using a prospective approach, the characteristics of post-acute COVID-19 patients of the different waves who participated in inpatient PR were compared based on their assessments and results collected as part of PR (Cumulative Illness Rating Scale (CIRS), six-minute walk test (6-MWT), Pulmonary Function Testing (PFT), and Functional Independent Measurement (FIM). (3) Results: A total of 483 patients were included in the analysis (Wave 1 n = 51, Wave 2 n = 202, Wave 3 n = 84, Wave 4 n = 146). Compared to Wave 3 + 4, patients of Wave 1 + 2 were older (69 vs. 63 years; *p* < 0.001), had a significantly lower CIRS (13.0 vs. 14.7 points; *p* = 0.004), had significant better PFT (FVC: 73 vs. 68%pred; *p* = 0.009; DLCO_SB_: 58 ± 18 vs. 50 ± 17%pred; *p* = 0.001), and showed significantly more comorbidities (2.0 vs. 1.6 n/pers.; *p* = 0.009). Wave 3 + 4 showed significantly greater improvements according to the 6-MWT (147 vs. 188 m; *p* < 0.001) and the FIM (5.6 vs. 21.1 points; *p* < 0.001). (4) Conclusions: Patients of the COVID-19 infection waves differed significantly according to their anthropometric data, incidence of comorbidities, and impact of the infection. All cohorts achieved clinically relevant and significant functional improvements during PR, with significant higher improvements in Wave 3 + 4.

## 1. Introduction

The COVID-19 pandemic in Switzerland started in the beginning of February 2020 as a regional sub-happening of the global outbreak of the respiratory disease COVID-19 and was based on infections with the SARS-CoV-2 virus, which had emerged in late 2019. The COVID-19 pandemic spread from the Chinese metropolis of Wuhan, Hubei Province, starting in December 2019. Beginning on 11 March 2020, the World Health Organization (WHO) classified the outbreak of the novel coronavirus as a pandemic [1]. The number of people infected with coronavirus in Switzerland has been increasing since the end of February 2020. Up until 12 December 2022, there were approximately 4.35 million confirmed cases of the disease in Switzerland. Due to or with co-occurring lung disease, more than 13,700 people have died in Switzerland so far.

During the pandemic, epidemiologists distinguished different waves of the spread that led to hospitalization of different population groups. The first wave occurred in spring 2020, the second period at the end of summer and in autumn 2020, and the third at the beginning of 2021. In Switzerland, a fourth wave started in October 2021 and lasted until June 2022 [2].

COVID-19 is caused by the severe acute respiratory syndrome coronavirus-2 (SARS-CoV-2). The pathophysiologic mechanism of this virus is mainly related to acute respiratory distress syndrome (ARDS) and systemic severe inflammatory reaction [3]. One of the first observations at the beginning of the pandemic was that patients with comorbidities, including previous cardiovascular disease, were more likely to present with worse clinical outcomes, including a higher risk of hospitalization and death [4,5]. Accordingly, the first three waves mainly affected people with severe courses of the disease who were already severely ill in advance. However, after an effective vaccine became available, the composition of the COVID-19 cohort with severe courses changed: those who had not been vaccinated predominated [6]. 

Pulmonary rehabilitation (PR) has been prioritized in COVID-19 patient care from the beginning [7,8]. Not only has it been used to provide patients with the usual rehabilitative services to promote recovery following a severe course, but in some countries (e.g., Switzerland, Germany, and Italy), it also, in part, has been used to relieve the acute hospitals and maintain their admission facilities and inpatient capacities [9].

Little has been described about the impact of changes in the composition of patients in the post-COVID-19 stage on rehabilitation outcomes, which is why, in this study, we analyzed the commonalities and differences in PR outcomes of the different patient groups.

## 2. Materials and Methods

Between February 2020 and June 2022, 676 patients with post-acute COVID-19 were admitted to an inpatient PR program at the Zurich RehaCenter Wald Clinic in Switzerland. For several reasons, data for only 483 patients could be included (Wave 1: 51, Wave 2: 202, Wave 3: 84, Wave 4: 146). Data collection was conducted prospectively. All included patients gave their written informed consent. For data storage, the clinic information system INES^®^ (INES Schweiz GmbH, Bottighoferstrasse 6, 8596 Scherzingen, Switzerland) was used. The study protocol was approved by the local ethics committee (BASEC-No 2020-01061) and was registered at the German Clinical Trials Register (DRKS00024613). 

All necessary assessments were performed upon admission to PR and 1–2 days before discharge. The contents of the assessments consisted of the Hospital Anxiety and Depression Scale (HADS), laboratory values, the Chronic Respiratory Disease Questionnaire (CRQ), the Cumulative Illness Rating scale (CIRS), and pulmonary function testing (PFT). The following assessments were performed twice, once upon admission and the second time upon discharge, in order to record changes: Feeling Thermometer (FT), Functional Independence Measure (FIM), and 6-min walk test. 

### 2.1. Pulmonary Rehabilitation Program

The contents of the PR program are described extensively elsewhere and are thus described only cursorily at this point [7].

All patients participated in an inpatient PR program of 3 weeks on average. The various sessions included individualized training, consisting of gymnastics and stretching, treadmill training or ergometer training, and walking training on terrain or indoors (three different levels of intensity), as well as strength training adapted to the performance capacity of the patients. The intensity level was based on the severity of the cardio-pulmonary effects as well as the functional limitations of the patients. 

Many patients showed significant limitations at the beginning of PR, so it was usually necessary to start with a low intensity level and successively increase the load, depending on the patient’s condition and tolerance. The gymnastic exercises addressed not only an improvement in endurance performance but also coordination and strength exercises, as well as exercises that trained flexibility and balance. Respiratory physiotherapy included cough control exercises in addition to breathing control and energy conservation techniques. All patients participated in educational sessions and nutritional interventions. If needed, patients participated in a structured smoking cessation program, received psychosocial support, or received diabetes counseling.

### 2.2. Six-Minute Walk Test (6-MWT)

Exercise capacity was measured at hospital admission and discharge using the 6-MWT according to the guidelines of the American Thoracic Society (ATS) and carried out by experienced, well-instructed examiners [10]. According to the ATS, this exercise test provides valuable data for patients with cardiac or pulmonary diseases, representing functional, therapeutic response as well as prognostic data. Due to its reproducibility and simplicity, the 6-MWT is frequently used and delivers a good overview of the cardiopulmonary and musculoskeletal status. This test is safe and well-tolerated by most patients at any stage of disease, with the test being highly reflective of usual daily activity and exercise performance.

### 2.3. Chronic Respiratory Disease Questionnaire (CRQ)

To assess health-related quality of life (HRQoL) the Chronic Respiratory Disease Questionnaire (CRQ) was used. The questionnaire measures eight dimensions of HRQoL and allows for the calculation of two summary scales of physical and mental experience [11]. The 20 items were completed by the patients individually. They represent the four areas of dysfunction (fatigue, emotional functioning, mastery, and dyspnea) in patients with chronic pulmonary diseases. The patients graded their symptoms according to a 7-point Likert scale for the domains, which included 4–7 items.

### 2.4. Functional Independence Measure (FIM)

The Functional Independence Measure (FIM) is an 18-item measurement tool that explores an individual’s physical, psychological, and social functioning. In this study, the FIM was used to assess changes in patient functioning, specifically with the aim of evaluating response to rehabilitation or medical intervention. The FIM uses level of assistance and individual needs to grade functional status from total independence to total support [12].

### 2.5. Hospital Anxiety and Depression Scale (HADS)

The HADS is questionnaire of 14 items (7 questions focussing on depression and 7 questions on anxiety). Scores range from 0 to 21, with higher scores representing greater disruption. Additionally, the questionnaire can be used as a diagnostic tool to assess depression in patient with severe physical restrictions [13]. Completion took about 2–5 min. In this study, the HADS was used to evaluate the response of depression and anxiety to PR. 

### 2.6. Cumulative Illness Rating Scale (CIRS)

The CIRS was used to assess a patient’s level of disability and as an indicator of health status, including predicted 18-month mortality and social function [14]. It is a comprehensive method for recording diseases in 14 organ systems on the basis of an evaluation of 0 to 4 points, which is used to calculate a cumulative score. The range of the total score is 0–56 points. When evaluating the CIRS, each individual illness in the corresponding organ system must be classified. If there were different diseases within the same organ system, only the disease that was most pronounced was evaluated. The calculated CIRS at admission is useful for predicting important hospital outcomes such as high risk of death or long stays and to better anticipate end-of-life issues.

### 2.7. Feeling Thermometer (FT)

The FT analyzes and evaluates the emotional status of the patients regarding their current well-being. Therefore, the patients rate their feelings according to a rating scale in terms of degrees from 0–100, representing their mood corresponding to temperatures [15]. A higher temperature indicates a better mood.

### 2.8. Pulmonary Function Tests (PFT) and Blood Gas Analysis

Upon each patient’s discharge from PR, we conducted comprehensive pulmonary functioning testing using Body-Plethysmography and Spirometry (Master Screen Body; Jaeger GmbH, Hoechberg, Germany). The tests were carried out considering the current ATS-ERS Guidelines. The following blood tests were analyzed once upon admission: arterial blood gas analysis (inhouse analysis: Radiometer ABL800, Willich, Germany) [16,17], blood cell counts, leukocytes, and C-reactive protein (CRP) (external laboratory analysis: Medica, Medizinische Laboratorien Dr. F. Kaeppeli AG, Zurich). 

### 2.9. Statistical Analysis

Binary variables were presented as relative and absolute frequencies, and Fisher’s exact test was used for group comparisons. Continuous variables were visually inspected using a Q-Q plot to verify normal distribution. Differences between groups in normally distributed variables were analyzed using one-way ANOVA with Tukey’s HSD as a post hoc test. To calculate the difference between groups of non-normally distributed data, the Kruskal-Wallis test with Dunn’s test and the Bonferroni correction as a post hoc test was used.

Using combined data across all waves, multivariable regression was used to determine variables independently associated with rehabilitation outcomes ∆6-MWT and ∆FIM. To ensure no multicollinearity between independent variables, the variance inflation factor (VIF) was calculated, and variables with values > 5 were further investigated for multicollinearity and not used in the regression model. Continuous variables describing patient’s characteristics were entered into the multivariable regression model, and non-significant variables were eliminated individually using backward subtraction.

## 3. Results

Out of 676 patients admitted to PR following a severe COVID-19 infection, 483 were included in the study. A total of 193 patients could not be considered for the following reasons: 98 refused to participate in the study, 55 did not complete the PR program for several reasons, 26 patient records were not completed, especially the assessments, and 14 did not participate for to other reasons. 

### 3.1. Patient Characteristics at Admission to Pulmonary Rehabilitation

Table 1 represents the baseline characteristics of all cohorts separated by COVID waves 1–4 upon admission to pulmonary rehabilitation. 

### 3.2. Independent Variables Predicting Pulmonary Rehabilitation Outcomes

Using combined baseline characteristics (age, BMI, acute hospital days, ICU days, ventilation days, CIRS, CRQ, 6-MWTpre, FIMpre, FTpre, HADS-A, HADS-D, FVC (pred%), FEV_1_%FVC, DLCO_SB_ (pred%), CRP, Hb, Leukocytes) and duration of PR across all waves, a multivariable regression was performed to determine the variables that independently predict PR outcomes, defined as ∆6-MWT, ∆FIM, and ∆FT. All independent variables showed a VIF < 5 with exception of ICD days and ventilation days, which correlated with each other (R = 0.94), hence they have not been used in combination in the regression model. In a multivariable model for ∆6-MWT, duration of PR, age, BMI, 6-MWTpre and HADS-D were independent predictors. For ∆FIM, duration of PR, age, and FIMpre turned out to be independent predictors. ∆FT is best predicted with the number of ventilation days and Ftpre. Results of the multivariable regression model are presented in Table 2.

Based on the multiple regression model, we conclude that age and duration of PR are the most important independent factors in determining overall PR outcome. When looking at the baseline characteristics by waves (Table 1), we see that age was significantly different between waves. However, Wave 1 + 2 (68.5 vs. 69.3 years; *p* = 0.975) and Wave 3 + 4 (63.6 vs. 62.3 years; *p* = 0.860) showed no significant difference between groups. Additionally, PR duration was not different between waves (*p* = 0.063). Furthermore, at Wave 1 + 2, no vaccination was available (vaccinated 0%), whereas starting in Wave 3 (vaccinated 8.3%), vaccinated patients began entering PR. Based on these analyses and to simplify further analysis and interpretation of data, we decided to compare Wave 1 + 2 to Wave 3 + 4 in further analysis.

### 3.3. Wave 1 + 2 versus Wave 3 + 4 Patients Characteristics Prior to COVID-19 Infection

Table 3 shows the patients’ characteristics and comorbidities prior to COVID-19 infection of Wave 1 + 2 and Wave 3 + 4. Patients from Wave 1 + 2 were older and had significantly lower CIRS and significantly better PFT. However, functional parameters (6-MWT, FIM, FT) at admission were not different. We found significantly more patients in Wave 1–2 having COPD, arterial hypertension, and atrial fibrillation. All other comorbidities were equally distributed. On average, Wave 1 + 2 showed significantly more comorbidities per patient compared to Wave 3 + 4.

### 3.4. Complications Due to COVID-19 Infection and Pulmonary Rehabilitation Outcome

Table 4 shows the incidence of newly acquired diseases or complications due to COVID-19 infection. We found significantly more lung infiltration, delirium, myocarditis, and renal insufficiency in the Wave 1 + 2 group, while hospital germs, venous thromboembolism (VTE), and pulmonary embolism occurred more often in the Wave 3 + 4 group. However overall complications per patient was not different between groups. 

Rehabilitation outcomes improved significantly in both groups (Wave 1 + 2 pre vs. post: 6-MWT: 190.4 vs. 338.8 m; *p* < 0.001; FIM: 94.7 vs. 111.0 points; *p* < 0.001; Wave 3 + 4 pre vs. post: 6-MWT: 183.6 vs. 367.7 m; *p* < 0.001; FIM: 91.9 vs. 112.6 points; *p* < 0.001). However, improvements compared between Wave 1 + 2 and Wave 3 + 4 according to the 6-MWT and FIM total scores were significantly higher in Wave 3 + 4, while PR duration, oxygen dependency at the end of PR, and FT were not different between Waves (Table 5).

## 4. Discussion

To the best of our knowledge, this is the first study with detailed characterization of patients from waves 1–4 of the coronavirus pandemic describing the impact on outcomes of an inpatient PR program. Patients differed significantly according to their anthropometric data, incidence of comorbidities, and impact of the infection. All patient groups achieved clinically relevant and significant functional improvements during PR, with significantly higher improvements in Wave 3 + 4. 

Patients of Wave 3 + 4 were significantly younger than patients of Wave 1 + 2. A reason for this might be the fact that the percentage of vaccinated persons in Switzerland as of 1 March 2021 increased with age, leading to more protection of elderly patients in Wave 3 + 4. In our study, only 29.1% of Wave 4 patients were vaccinated, while during the same period, the vaccination status of the population of Switzerland had already risen up to 71.7% [18]. As a result, we saw younger unvaccinated and severely ill patients in Wave 3 + 4. In an observational study regarding a similar time period (October 2018 to September 2021), Sleffel et al. found in a comparable age structure in 1520 PR participants [19]. The vaccination status might have led to this shift in age, but the awareness of elderly people at risk, the protection measures available, and, in some cases, initial infection during Wave 1 + 2 can potentially be additional factors. 

Probably due to the age differences, clinically relevant comorbidities such as COPD, arterial hypertension, or atrial fibrillation were significantly more frequent in Wave 1 + 2 than in Wave 3 + 4. The patient characteristics of Wave 1 + 2 are comparable to the findings in other cohorts of the same time period, which also had numerous comorbidities and were even older on average (72 years) [20].

It is well known that patients with pulmonary diseases show improvements in performance by participating in PR, e.g., regarding the walking distance according to the 6-MWT [21]. Functional benefits of PR participation in post-COVID-19 patients have been published previously by our group [7]. In our present study, patients in all groups showed significant improvements according to the assessments, and the enhancements are comparable to results in other studies (6-MWT 132.8 ± 92.8 m; FIM 18.0 ± 11.4 points) [22]. In the intergroup comparison of Wave 1 + 2 and Wave 3 + 4, however, there was a significantly and clinically relevant higher improvement found for the 6-MWT and FIM in patients from Wave 3 + 4. As the multivariable model shows, duration of PR, age, and the functional level (6-MWT and FIM) at admission are the major predicting factors for the PR outcome. BMI and HADS-D also play a role, but only for the 6-MWT improvement. The model shows that younger and highly impaired COVID patients with a long PR duration are more likely to benefit from PR. However, ICU days and hospitalization duration showed no further influence on the PR outcomes, while ventilation days positively affected the ∆FT only. These findings are in line with previous studies [23]. 

Gabunia S et al. compared 138 patients from the first two waves of the COVID-19 pandemic according to the results achieved while participating in inpatient PR with a length of stay of 11–12 days. The patients in both waves experienced the same functional improvements regarding the GG Self-care and Mobility Activities items with a median GG score change of 3.6 per day and similar discharge GG scores [24]. However, the patients of the respective waves did not differ significantly, and enhancements during PR were similar. These findings are in line with our results, showing no difference in patient characteristics followed by almost the same PR outcomes. However, our data show that as soon as the vaccination status changed, which was found in Wave 3 + 4, patient characteristics also changed, leading to a positive impact on PR outcome parameters.

It has been explained that COVID-19 infection has numerous manifestations beyond the respiratory system, including cardiac injury (cardiomyopathy, myocarditis, ventricular arrhythmias, and hemodynamic instability in the absence of obstructive coronary artery disease) [25]; thrombotic complications (including stroke, myocardial infarction, and venous thromboses) [26]; and renal, gastrointestinal, and neurologic symptoms, among others [27]. These manifestations are frequently followed by persistence of symptoms and a reduced quality of life [28]. This is in contrast to our study, wherein the more symptomatic and impaired patient group of Wave 3 + 4 showed better PR outcomes. 

However, regarding the well-being of the patients measured by the FT, no significant differences between the groups were observed. This discrepancy is in contrast to the results described by Jacobs LG et al., who found a large amount of prevalent and persistent symptoms according to their observational study and found that the persistence of symptoms has an important impact on general, physical, and mental health status, social functioning, and quality of life within 35 days of discharge after COVID-19 infections [29]. We assume that the perception of the enhancements while participating in the PR program was mainly reduced due to mood disorders following the COVID-19 infection, though the HADS-D scores were significantly higher in Wave 1 + 2. However, the experience in treating the patients of Wave 3 + 4 was that they noted dissatisfaction with their situation in general and that the expectations projected in the PR were not fulfilled. 

As already described, severe COVID-19 infections may lead to persisting lung function impairment, especially regarding the diffusion capacity (DLCO) [30]. The findings of our analysis confirm the results published by Lenoir A et al. showing a reduction of the DLCO (53.8%pred.) at admission to PR in all waves. Additionally, persistent oxygen dependency was observed in 54.1% of our cohort at discharge from PR. This indicated the severity of the lung damage caused by the COVID-19 infection, which seemed to be higher than that found in other cohorts. The chest x-rays performed at discharge from PR showed significant more pulmonary infiltrates for the Wave 1 + 2 patients. According to recent studies, the reported quota of oxygen dependency after COVID 19-infection differs to a huge extent depending on the observed cohort. For example, Jacobs LG et al. reported 20.3% oxygen dependence 35 days after COVID-19 infection, while Sundh J et al. described 67% following 150 days of the infection [29,31]. It seems to be obvious that the quota depends on the initial severity of the infection and the number of pulmonary comorbidities. However, most of those with abnormal pulmonary function tests at 3 months improved subsequently, but only another 29% (6 out of 21) reached normal values at 6 months [32].

This analysis has some limitations, which have to be discussed. First, this study represents data from a single center. Therefore, results should be applied to other cohorts with caution. However, our rehab center is one of the largest centers in Eastern Switzerland, and the area includes many different acute hospitals that referred patients to PR. This is why we believe that the cohort is representative of patients with severe post-acute COVID-19 infections. Second, since the approach to the COVID-19 patients in this study is observational, it provides no control group. Implementation of a control group was not feasible in this context for legal and ethical reasons. On the one hand, patients have a right to rehabilitative services, and on the other hand, withholding rehabilitation would not be ethically defensible or enforceable in Switzerland. Third, a potential selection bias may have occurred due to the amount of patient data that could not be considered, as described in the results section.

## 5. Conclusions

The study shows that participation in PR led to significant improvements in performance, well-being, and functionality in both Wave 1 + 2 and Wave 3 + 4. However, the enhancements in performance and functionality were significantly higher in Wave 3 + 4. We hypothesize that the higher vaccination status during Wave 3 + 4 changed the patient characteristics towards lower age, hence fewer morbidities and greater impairment than during previous waves, which lead to an improved PR outcome.

## Figures and Tables

**Table 1:** Patient characteristics upon admission to pulmonary rehabilitation.

	Wave 1 (n = 51)	Wave 2 (n = 202)	Wave 3 (n = 84)	Wave 4 (n = 146)	*p*
Age [year ± SD]	68.5 ± 9.1	69.3 ± 10.9	63.6 ± 11.2 ^$^	62.3 ± 13.3 *^,$^	<0.001
Gender, female [%, (n)]	39.2(20)	33.7(68)	38.6(32)	32.2(47)	0.630
BMI [kg/m^2^ ± SD]	27.6 ± 6.2	27.2 ± 10.9	26.6 ± 4.2	27.3 ± 5.4	0.673
Vaccinated [%(n)]	0(0)	0(0)	8.3(7)	21.9(32) *^,$,#^	<0.001
Acute hospital days [d ± SD]	29.5 ± 17.3	21.7 ± 12.7 *	26.8 ± 19.2	22.8 ± 12.1	0.003
ICU days [d ± SD]	14.3 ± 13.0	6.6 ± 10.8 *	10.5 ± 18.6	9.4 ± 13.6	0.002
Ventilation days [d ± SD]	9.8 ± 10.3	4.1 ± 8.7 *	6.2 ± 14.0	6.1 ± 11.5	0.007
O2 Therapy pre [%(n)]	52.9(27)	40.6(82)	46.4(39)	41.8(61)	0.392
CIRS [points ± SD]	14.4 ± 5.8	12.6 ± 6.4	14.0 ± 6.2	15.1 ± 6.8 ^$^	0.006
CRQ [points ± SD]	4.7 ± 1.0	4.7 ± 1.0	4.7 ± 1.2	4.7 ± 1.2	0.979
6-MWTpre [m ± SD]	185.1 ± 151.2	192.0 ± 129.7	187.6 ± 132.5	181.3 ± 132.5	0.918
FIMpre [points ± SD]	101.6 ± 14.3	92.9 ± 17.9 *	92.5 ± 18.9 *	91.6 ± 13.8 *	0.003
FTpre [points ± SD]	51.5 ± 15.7	54.1 ± 17.4	48.4 ± 16.9	53.7 ± 19.9	0.173
HADS-A [points ± SD]	5.3 ± 3.9	6.4 ± 8.1	5.1 ± 4.1	4.4 ± 3.7	0.066
HADS-D [points ± SD]	5.0 ± 2.9	6.4 ± 3.5	4.9 ± 3.6 ^$^	4.8 ± 4.1 ^$^	0.005
FVC [pred% ± SD]	74.8 ± 18.8	73.1 ± 20.0	70.2 ± 18.7	66.9 ± 18.3	0.042
FEV_1_/FVC [% ± SD]	79.8 ± 9.4	79.4 ± 11.3	82.7 ± 8.5	81.3 ± 10.1	0.174
DLCO_SB_ [pred% ± SD]	58.4 ± 16.4	57.4 ± 18.2	51.1 ± 19.0	49.8 ± 15.3 ^$^	0.005
CRP [mg/L ± SD]	161.2 ± 145.5	126.9 ± 98.2	153.5 ± 115.1	106.7 ± 89.5 *^,#^	<0.001
Hb [G/L ± SD]	94.5 ± 22.8	108.5 ± 21.6 *	109.2 ± 20.0 *	114.7 ± 19.8 *^,$^	<0.001
Leukocytes [G/L ± SD]	12.2 ± 7.3	12.3 ± 6.0	12.3 ± 6.3	11.3 ± 5.0	0.417
COPD [%(n)]	13.7(7)	9.4(19)	3.6(3)	5.5(8)	0.091
Alcohol [%(n)]	3.9(2)	6.9(14)	7.1(6)	8.2(12)	0.784
CHD [%(n)]	17.6(9)	17.3(35)	14.3(12)	16.4(24)	0.931
CHF [%(n)]	2.0(1)	3.0(6)	2.4(2)	1.4(2)	0.801
Pul. hypertension [%(n)]	0.0(0)	1.0(2)	0.0(0)	1.4(2)	0.635
Art. hypertension [%(n)]	52.9(27)	55.0(111)	41.7(35)	37.0(54) ^$^	<0.001
Diabetes [%(n)]	27.5(14)	27.2(55)	26.2(22)	26.0(38)	0.993
PAD [%(n)]	7.8(4)	5.9(12)	7.1(6)	6.8(10)	0.956
A-fib [%(n)]	7.8(4)	13.9(28)	6.0(5)	2.8(4) ^$^	0.002
Stroke [%(n)]	2.0(1)	5.9(12)	4.8(4)	6.2(9)	0.627
DVT [%(n)]	2.0(1)	2.5(5)	4.8(4)	0.7(1)	0.257
PE [%(n)]	2.0(1)	3.0(6)	3.6(3)	4.1(6)	0.880
Dyslipidemia [%(n)]	17.6(9)	17.8(36)	15.8(13)	13.0(19)	0.652
Mental disorders [%(n)]	11.7(6)	9.4(19)	13.1(11)	10.3(15)	0.815
Renal insufficiency [%(n)]	23.5(12)	19.3(39)	17.9(15)	11.0(16)	0.103
Comorbidities [n/pers ± SD]	1.9 ± 1.6	2.0n ± 1.6	1.7 ± 1.7	1.5 ± 1.7 ^$^	0.010

BMI, body mass index; ICU, intensive care unit; CIRS, Cumulative Illness Rating Scale; CRQ, Chronic Respiratory Disease Questionnaire; 6-MWT, 6-min walk test; FIM, Functional Independence Measure; FT, feeling thermometer; HADS-A, Hospital Anxiety and Depression Scale—Anxiety; HADS-D, Hospital Anxiety and Depression Scale—Depression; FVC, functional vital capacity; FEV1, forced expiratory pressure in 1 s; DLCOSB, carbon monoxide diffusion capacity of the lung; CRP, c-reactive protein; Hb, hemoglobin, COPD, chronic obstructive pulmonary disease; CHD, coronary heart disease; CHF, chronic heart failure; PAD, peripheral artery disease; A-fib, atrial fibrillation; DVT, deep vein thrombosis; PE, pulmonary embolism, * vs. Wave 1; ^$^ vs. Wave 2; ^#^ vs. Wave 3.

**Table 2:** Multivariable regression model.

	*B*	Wald 95% CI	*p*
Independent variables predicting ∆6-MWT			
Duration of PR	2.88	2.19–3.57	<0.001
Age	−3.01	−3.47–−2.55	<0.001
BMI	−3.87	−4.86–−2.88	<0.001
6-MWTpre	−0.35	−0.39–−3.15	<0.001
HADS-D	−4.14	−5.55–−2.73	0.004
Independent variables predicting ∆FIM			
Duration of PR	0.35	0.31–0.39	<0.001
Age	−0.25	−0.29–−0.21	<0.001
FIMpre	−0.42	−0.45–−0.39	<0.001
Independent variables predicting ∆FT			
Ventilation days	0.21	0.13–0.29	0.011
Ftpre	−0.65	−0.68–−0.61	<0.001

6-MWT, 6-min walk test; FIM, Functional Independence Measurement; FT, feeling thermometer; PR, pulmonary rehabilitation; BMI, body mass index; HADS-D, Hospital Anxiety and Depression Scale—Depression; ICU, intensive care unit.

**Table 3:** Wave 1 + 2 versus Wave 3 + 4 patient characteristics prior to COVID-19 infection.

	Wave 1 + 2 (n = 253)	Wave 3 + 4 (n = 230)	*p*
Age [year ± SD]	69.1 ± 10.5	62.8 ± 12.6	<0.001
CIRS [points ± SD]	13.0 ± 6.3	14.7 ± 6.6	0.004
6-MWTpre [m ± SD]	190.4 ± 134.5	183.6 ± 132.5	0.592
FIMpre [points ± SD]	94.7 ± 17.5	91.9 ± 15.9	0.074
FTpre [points ± SD]	53.5 ± 17.0	51.8 ± 19.1	0.364
FVC [pred% ± SD]	73.4 ± 19.7	68.0 ± 18.5	0.009
DLCO_SB_ [pred% ± SD]	57.6 ± 17.8	50.3 ± 16.7	0.001
COPD [%(n)]	10.3(26)	4.8(11)	0.026
Alcohol [%(n)]	6.3(16)	7.8(18)	0.595
CHD [%(n)]	17.4(44)	15.7(36)	0.930
CHF [%(n)]	2.8(7)	1.7(4)	0.549
Pul. hypertension [%(n)]	0.8(2)	0.9(2)	1.000
Art. hypertension [%(n)]	54.5(138)	38.7(89)	<0.001
Diabetes [%(n)]	27.3(69)	26.1(60)	0.837
PAD [%(n)]	6.3(16)	7.0(16)	0.856
A-fib [%(n)]	12.6(32)	3.9(9)	<0.001
Stroke [%(n)]	5.5(14)	5.7(13)	1.000
DVT [%(n)]	2.4(6)	2.2(5)	1.000
PE [%(n)]	2.8(7)	3.9(9)	0.613
Dyslipidemia [%(n)]	17.8(45)	13.9(32)	0.264
Mental disorders [%(n)]	9.9(25)	11.3(26)	0.658
Renal insufficiency [%(n)]	20.2(51)	13.5(31)	0.053
Comorbidities [n/pers ± SD]	2.0 ± 1.6	1.6 ± 1.7	0.009

CIRS, Cumulative Illness Rating Scale; 6-MWT, 6-min walk test; FIM, Functional Independence Measure; FT, feeling thermometer; FVC, functional vital capacity; DLCOSB, carbon monoxide diffusion capacity of the lung; COPD, chronic obstructive pulmonary disease; CHD, coronary heart disease; CHF, chronic heart failure; PAD, peripheral artery disease; A-fib, atrial fibrillation; DVT, deep vein thrombosis; PE, pulmonary embolism.

**Table 4:** Newly acquired diseases and complications caused by COVID-19 infection.

	Wave 1 + 2 (n = 253)	Wave 3 + 4 (n = 230)	*p*
Lung infiltration [%(n)]	83.0(210)	70.0(161)	<0.001
COPD [%(n)]	0.8(2)	0.0(0)	0.500
Hospital germs [%(n)]	2.0(5)	7.4(17)	0.007
Sepsis [%(n)]	27.7(70)	24.3(56)	0.468
Delirium [%(n)]	29.2(74)	19.1(44)	0.011
CHD [%(n)]	0.8(2)	1.7(4)	0.431
CHF [%(n)]	3.6(9)	2.2(5)	0.425
Pulmonary hypertension [%(n)]	1.2(3)	1.3(3)	1.000
Arterial hypertension [%(n)]	3.6(9)	6.5(15)	0.147
Diabetes [%(n)]	5.5(14)	6.1(14)	0.847
PAD [%(n)]	0.0(0)	0.4(1)	0.435
A-fib [%(n)]	9.5(24)	9.1(21)	1.000
Stroke [%(n)]	3.0(21)	0.9(2)	1.000
DVT [%(n)]	1.2(3)	4.3(10)	0.046
Enteritis [%, (n)]	1.2(3)	0.4(1)	0.625
Myocarditis [%(n)]	5.1(13)	1.3(3)	0.022
ARDS [%(n)]	45.8(116)	42.6(98)	0.521
PE [%(n)]	9.9(25)	20.9(48)	<0.001
Dyslipidemia [%(n)]	2.0(5)	0.4(1)	0.219
Mental disorders [%(n)]	7.9(20)	8.7(20)	0.744
Renal insufficiency [%(n)]	27.7(70)	14.8(34)	<0.001
ICU-acquired weakness [%(n)]	11.9(30)	12.6(29)	0.890
PNP [%(n)]	11.1(28)	9.6(22)	0.655
Complications [n/pers ± SD]	2.9 ± 1.9	2.7 ± 1.7	0.115

COPD, chronic obstructive pulmonary disease; CHD, coronary heart disease; CHF, chronic heart failure; PAD, peripheral artery disease; A-fib, atrial fibrillation; DVT, deep vein thrombosis; ARDS, acute respiratory distress syndrome; PE, pulmonary embolism; PNP, polyneuropathy.

**Table 5:** Outcome parameters of the pulmonary rehabilitation program.

	Wave 1 + 2 (n = 253)	Wave 3 + 4 (n = 230)	*p*
Duration of PR [d ± SD]	21.4 ± 9.2	23.9 ± 17.1	0.050
Oxygen dependancy [%(n)]	69.6(176)	37.4(86)	0.251
∆6-MWT [m ± SD]	147.4 ± 105.8	188.1 ± 113.5	<0.001
∆FIM [points ± SD]	15.6 ± 12.6	21.1 ± 16.9	<0.001
∆FT [points ± SD]	20.8 ± 15.4	23.0 ± 18.7	0.263

PR, pulmonary rehabilitation; 6-MWT, 6-min walk test; FIM, Functional Independence Measurement; FT, feeling thermometer.

## Data Availability

Data supporting the reported results can be provided upon reasonable request by the corresponding author.
